# Polymyalgia Rheumatica Following Influenza B Infection: A Case Report

**DOI:** 10.7759/cureus.70671

**Published:** 2024-10-02

**Authors:** Koji Hayashi, Hiroaki Maeda, Hiromi Hayashi, Asuka Suzuki, Yuka Nakaya, Mamiko Sato, Kouji Hayashi, Yasutaka Kobayashi

**Affiliations:** 1 Department of Rehabilitation Medicine, Fukui General Hospital, Fukui, JPN; 2 Graduate School of Health Science, Fukui Health Science University, Fukui, JPN

**Keywords:** giant cell arteritis with polymyalgia rheumatica, influenza b, influenza virus, influenza virus type b, polymyalgia rheumatica

## Abstract

We describe a case of polymyalgia rheumatica (PMR) following an influenza B infection. The patient was a 71-year-old woman who developed a fever above 38.0°C and was diagnosed with influenza B, confirmed by a rapid antigen test. Although the fever resolved after three days, she continued to experience neck pain, back pain, and joint pain, particularly in both shoulders and hips. She also reported morning joint stiffness lasting for more than an hour and occasional low-grade fever. She presented to our hospital 25 days after the onset of symptoms. Blood tests revealed elevated C-reactive protein and erythrocyte sedimentation rate, but levels of creatine phosphokinase, rheumatoid factor, and anti-cyclic citrullinated peptide were not elevated. She was diagnosed with PMR and treated with prednisolone (PSL) 15 mg/day. The response to steroids was remarkably good, and PSL was tapered over six months. PMR is believed to result from an immune-mediated process and may be associated with certain human leukocyte antigen haplotypes. Additionally, PMR is sometimes preceded by an infection. To date, there have been very few reports suggesting a connection between influenza B and PMR, underscoring the need for further case accumulation.

## Introduction

Polymyalgia rheumatica (PMR) is an inflammatory condition characterized by pain and stiffness, primarily affecting the shoulders and pelvic region in individuals over 50 years of age [1]. Blood tests typically reveal elevated inflammatory markers, and symptoms usually improve with steroid treatment [1]. While the exact cause of PMR remains unclear, most research suggests a multifactorial origin leading to an immune-mediated disease process [2].

Influenza B virus, part of the *Betainfluenzavirus* genus, co-circulates annually with influenza A during seasonal epidemics and can cause severe respiratory illness in humans [3]. Unlike influenza A, influenza B lacks pandemic potential due to the absence of an animal reservoir, which may explain the relatively lower attention it receives [3,4]. However, influenza B still significantly contributes to the global flu burden, accounting for 23% of annual influenza cases [3,5]. To the best of our knowledge, although immune-mediated complications related to influenza B infections are limited, multiple reports exist on Guillain-Barré syndrome and acute necrotizing encephalopathy associated with influenza B infections [6-9]. However, reports of post-infectious PMR induced by influenza B are scarce, and its incidence remains unclear. In this report, we describe a rare case of PMR following influenza B infection.

## Case presentation

A 71-year-old woman developed a fever above 38.0°C and was diagnosed with influenza B, confirmed by a rapid antigen test. A COVID-19 antigen test was negative. She had a history of cerebral infarction at the age of 69, but she did not have any residual symptoms. Although the fever resolved after three days, she continued to suffer from neck pain, back pain, and joint pain, particularly in both shoulders and hips. She also reported morning joint stiffness lasting for more than an hour and occasional low-grade fever. The pain throughout her body caused sleep disturbances at night, and the stiffness made it difficult for her to move around in bed in the morning. Additionally, she had difficulty elevating her shoulders and flexing her hips and knees.

She visited both orthopedic and internal medicine clinics, but no diagnosis was made. She presented to our hospital 25 days after the onset of symptoms. Her vital signs were unremarkable, including a temperature of 36.7°C. She was able to walk normally, but pain in the muscles and joints of the neck, shoulders, back, and buttocks was noted. However, there was no tenderness upon palpation. Palpation also revealed no thickening or tenderness of the temporal artery. Blood tests revealed a significantly elevated CRP level of 3.87 mg/dl and ESR of 52 mm/hr, along with decreased white blood cell count (3000/μl), hemoglobin (11.3 g/dl), albumin (3.8 g/dl), sodium (137 mmol/l), and chlorine (99 mmol/l) (Table 1). Notably, rheumatoid factor (RF) and anti-cyclic citrullinated peptide (anti-CCP) levels were within normal ranges (Table 1).

**Table 1:** The results of blood tests on admission.

Inspection items	Result	Reference range
White blood cell count	3000/μl	3300–8600
Red blood cell count	396×10⁴/μl	386–492×10⁴
Hemoglobin	11.3 g/dl	11.6–33.4
Blood platelet	28.1×10⁴/μl	15.8–34.8
Total protein	7.1 g/dl	6.6–8.1
Albumin	3.8 g/dl	4.1–5.1
Glucose	97 mg/dl	73–109
Blood urea nitrogen	9.1 mg/dl	8.0–20.0
Creatinine	0.63 mg/dl	0.46–0.79
Total bilirubin	0.4 mg/dl	0.4–1.2
Aspartate aminotransferase	22 U/l	13–30
Alanine aminotransferase	25 U/l	7–30
Alkaline phosphatase	83 U/l	38–113
Lactate dehydrogenase	178 U/l	124–222
γ-glutamyltransferase	28 U/l	13–64
Creatine phosphokinase	45 U/l	41–153
Choline esterase	252 U/l	240–486
Amylase	55 U/l	44–132
Sodium	137 mmol/l	138–145
Potassium	4.0 mmol/l	3.6–4.8
Chlorine	99 mmol/l	101–108
Calcium	9.4 mg/dl	8.8–10.1
C-reactive protein	3.87 mg/dl	0.00–0.14
Erythrocyte sedimentation rate	52 mm/hr	<15 mm/hr
Rheumatoid factor	0.8 IU/ml	0.0–15.0
Anti-cyclic citrullinated peptide antibodies	<0.5 U/ml	0.0–4.4

We recommended further tests to investigate possible malignancy or infections, including contrast-enhanced computed tomography, gastrointestinal endoscopy, interferon-gamma release assay, blood cultures, and echocardiography. However, the patient declined and requested immediate treatment. We initiated prednisolone (PSL) at 15 mg/day, and her clinical symptoms, including pain and restricted range of motion, improved. The PSL dose was tapered every two weeks until it reached one mg/day over six months, at which point her symptoms worsened. Blood markers showed a slight increase, with CRP at 0.95 mg/dl and ESR at 24 mm/hr. The PSL dose was increased to 5 mg/day, and as of this writing, her clinical symptoms have resolved with PSL treatment.

## Discussion

We describe a rare case of PMR following an influenza B infection. For the diagnosis of PMR, the American College of Rheumatology (ACR)/European League Against Rheumatism (EULAR) 2012 criteria are commonly used [10]. Based on these criteria, our case met all three required conditions: age over 50 years, bilateral shoulder pain, and elevated CRP and/or ESR levels. Additionally, our case satisfied all four clinical criteria without the need for ultrasound, including morning stiffness lasting more than 45 minutes, hip pain or limited range of motion, RF and anti-CCP levels, and the absence of other joint pain. Moreover, the patient fulfilled Bird et al.'s diagnostic criteria for classic PMR [11]. Lastly, PMR typically responds exceptionally well to steroid treatment [10], and our case demonstrated a strong response to this therapy.

To our knowledge, several cases of post-infectious PMR have been reported in association with *Mycoplasma pneumoniae*, *Chlamydia* (*Chlamydophila*) *pneumoniae*, parvovirus B19, herpes zoster, and COVID-19 infections [12-17]. Aside from some previously reported cases being accompanied by giant cell arteritis (GCA), no other distinctive features were noted in comparison to our case [15,16]. Additionally, only one case of influenza B-related PMR has been documented [18]. We were unable to find any report of other influenza types including influenza A related to PMR. Furthermore, there have been reports of PMR and GCA following influenza vaccination, with research suggesting that the presence of one of the human leukocyte antigen (HLA) haplotypes, the DRB1*13:01 haplotype, may increase the risk of developing PMR/GCA post vaccination [19]. Other reports supported the relationship between HLA haplotypes such as HLA-DRB1 or HLA-DQB1 and PMR [20]. Based on these findings, although the detailed mechanisms behind PMR development following infectious diseases remain unclear, microbial antigens and specific HLA haplotypes may play a role in triggering PMR.

There are two major limitations. Firstly, we were unable to conduct several tests to exclude other conditions. PMR presents with nonspecific features that can be mimicked by numerous other entities. Differential diagnoses for PMR include crowned dens syndrome, hypothyroidism, obstructive sleep apnea, depression, viral infections (such as Epstein-Barr virus, hepatitis, HIV, and parvovirus B19), systemic bacterial infections, septic arthritis, cancer, diabetes, and remitting seronegative symmetrical synovitis with pitting edema (RS3PE) syndrome [21]. Our case met the diagnostic criteria, and the clinical course was consistent with PMR. However, further testing was necessary for a more accurate diagnosis.

The second limitation of this report is that it describes a single case, and the association between influenza B infection and PMR cannot be definitively established. Moreover, previous reports supporting these relationships are quite limited. However, we believe that the temporal proximity of symptom onset following the influenza B infection strongly suggests its contributory role in the pathogenesis of PMR. Further case accumulation is needed to substantiate this association.

## Conclusions

We present a case of PMR following an influenza B infection. Fever and muscle pain are common symptoms of influenza infection, but if they persist beyond the usual duration or reappear, PMR should be suspected. PMR is thought to result from an immune-mediated disease process and may be associated with certain HLA haplotypes. Additionally, PMR is sometimes preceded by an infection. To date, there have been very few reports suggesting a connection between influenza B and PMR, highlighting the need for further case accumulation.
